# Association between Tumorigenic Potential and the Fate of Cancer Cells in a Syngeneic Melanoma Model

**DOI:** 10.1371/journal.pone.0062124

**Published:** 2013-04-23

**Authors:** Yakov Krelin, Liron Berkovich, Moran Amit, Ziv Gil

**Affiliations:** 1 The Laboratory for Applied Cancer Research, Tel Aviv Sourasky Medical Center, Sackler Faculty of Health, Tel Aviv University, Tel Aviv, Israel; 2 Department of Otolaryngology Head and Neck Surgery, Rambam Medical Center, Rappaport School of Medicine, Technion Israel Institute of Technology, Haifa, Israel; Duke University Medical Center, United States of America

## Abstract

The self-renewal potential of a cancer cell can be estimated by using particular assays, which include xenotransplantation in immunocompromised animals or culturing in non-adherent serum-free stem-cells media (SCM). However, whether cells with self-renewal potential actually contribute to disease is unknown. Here we investigated the tumorigenic potential and fate of cancer cells in an in-vivo melanoma model. We examined cell lines which were derived from the same parental line: a non-metastatic cell line (K1735/16), a metastatic cell line (K1735/M4) and a cell line which was selected in non-adherent conditions (K1735/16S). All cell lines exhibited similar proliferation kinetics when grown on culture plates. K1735/16 cells grown in soft agar or in suspension non-adherent conditions failed to form colonies or spheroids, whereas the other cell lines showed prominent colonogenicity and spheroid formation capacity. By using sphere limiting dilution analysis (SLDA) in serum-free media, K1735/16S and K1735/M4 cells grown in suspension were capable of forming spheroids even in low frequencies of concentrations, as opposed to K1735/16 cells. The tumorigenic potential of the cell lines was determined in SCID mice using intra footpad injections. Palpable tumors were evident in all mice. In agreement with the *in-vitro* studies, the K1735/M4 cell line exhibited the highest growth kinetics, followed by the K1735/16S cell line, whereas the K1735/16 cell line had the lowest tumor growth potential (*P*<0.001). In contrast, when we repeated the experiments in syngeneic C3H/HeN mice, the K1735/16 cell line produced macroscopic tumors 30–100 days after injection, whereas K1735/M4 and K1735/16S derived tumors regressed spontaneously in 90–100% of mice. TUNEL analysis revealed significantly higher number of apoptotic cells in K1735/16S and K1735/M4 cell line-derived tumors compared to K1735/16 tumors (P<0.001). The models we have examined here raised the possibility, that cells with high-tumorigenic activity may be more immunogenic and hence are more susceptible to immune-regulation.

## Introduction

Cancer is a complex disease, involving differences between tumors or cells within a given tumor, as well as variation between patients. Within the spectrum of cells in a given tumor, subpopulations of cells may be phenotypically different and exhibit distinct proliferative potential. For example, the cancer stem cell (CSC) model suggests that only a small subpopulation of cells has a self-renewal and tumor formation potential, while the majority of the tumor consists of non-tumorigenic cells [1]. Evidence supporting the CSC model is found in germ cell cancer, leukemia, breast cancer, colon cancer and in some brain cancers. [2], [3], [4], [5], [6], [7], [8], [9], [10], [11]. On the other hand, whether melanomas are consistent with such a model is a matter of continuous debate [12], [13].

Currently, the only assay that determines the tumorigenic potential of human tumors involves xenotransplantation of different subpopulations of cancer cells into flanks of highly immunosuppressive animals (e.g. NOD/SCID mice). In addition, stemness (i.e. the ability to self-renew and differentiate) is frequently evaluated *in-vitro* by surrogate assays that examine the sphere-forming ability and clonogenicity in anchorage independent conditions, such as semisolid soft agar [14]. Previous experiments showed that multicellular tumor spheroids are morphologically and characteristically similar to solid tumours *in-vivo* [15], [16]. It has also been demonstrated that the sphere-forming potential in suspension non-adherent conditions consistently correlates with the neoplastic growth potential in immunosuppressed mice [17], [18], [19], [20].

Both *in-vitro* and *in-vivo* stemness assays address the tumorigenic potential of distinct subpopulation of cells, whereas the actual formation of tumors in patients may depend on other factors. The tumor microenvironment that may be site specific and the host immune system that is impaired in NOD/SCID mice can potentially alter the fate of cancer cells and their contribution to the disease. Hence, the question of whether cells with a high tumorigenic potential actually contribute to the tumor growth in patients with an intact immune system remains unresolved.

In this paper we sought to compare two phenomena related to cancer development: tumorigenic potential and the fate of cancer cells. To overcome two of the main limitations that are inherent to subcutaneous xenografting of human cancer cells into immunocompromised mice, i.e. the species barrier and the transplantation setting, we used a syngeneic melanoma model and orthotopic intra footpad injections into immune-competent animals.

## Materials and Methods

### Cell Lines

Mouse melanoma cell lines (K1735/16 and K1735/M4) were a gift from the laboratory of Dr. Lea Eisenbach (the Weizmann Institute, Rehovot). The K1735/16S cell line was derived from the K1735/16 cell line, by culturing cells in non-adherent conditions (see below) for 16 days. Cells were grown in DMEM supplemented with MSCM, 100 U/ml penicillin and 100 µg/ml streptomycin, at 37°C, 5% CO2, in a humidified incubator. All medium ingredients were purchased from Biological Industries, Israel. For self renewal and spheroid growth assays we used melanoma serum-free stem cell media (MSCM) that consisted of Dulbecco’s modified Eagle’s medium/F12, KnockOut™ SR, 100 mM L-glutamine (Invitrogen), MEM Non-Essential Amino Acids Solution 10 mM, 2 µg/ml FGF (Sigma), and antibiotics. For sphere growth assays we used MSCM conditioned with mouse embryonic fibroblasts (MEF) CF-1 for 24 h. [21] Also were used reagents such as: sodium azide, paraformaldehyde, xylene and sodium citrate were purchased from Sigma Aldrich, Israel.

### Mice and the *in vivo* Foot Pad Model

Female C3H/HeN mice and Severe Combined Immunodeficiency (SCID) mice were purchased from Harlan (Jerusalem, Israel). All mice were kept at the Animal Facilities of the Tel Aviv Medical Center (Tel-Aviv, Israel), under aseptic conditions.

Animal studies were performed in compliance with all applicable policies, procedures and regulatory requirements of the Institutional Animal Care and Use Committee (IACUC), the Research Animal Resource Center (RARC) of Tel Aviv University and the National Institutes of Health (NIH) “Guide for the Care and Use of Laboratory Animals”. All animal procedures were performed by inhalation of 2% isoflurane. After the studies, all animals were sacrificed by CO_2_ inhalation.

A foot pad syngeneic melanoma model was established, as described previously by Harrell et al. [22]. Briefly, thirty, 6-week-old mice were anesthetized with inhalational isoflurane for all procedures. The left hind limb foot pad was sterilized with alcohol and then slowly injected with 50 µL of cell suspension at a concentration of 2×10^5^ cells/50 µL over a 2-minute period. The mice were then awakened and their foot pad monitored for tumor size and signs of pain or ulceration twice a week.

Experiment with syngeneic C3H/HeN mice, were repeated 3 times, and in each experiment we injected 10–15 mice per group. In the experiments with SCID mice we used 6–7 mice per group. In the cell sorting experiment, each group (5–6 mice per group) was injected with sorted CD133(+), control K1735/M4 or control K1735/16 cells to syngeneic C3H/HeN mice.

### Immunohistochemistry

Samples from the injection site of melanoma cell lines were obtained on days 16 and 40, fixed in 4% paraformaldehyde, dehydrated in alcohol, cleared in xylene and embedded in paraffin. Four-micron sections were stained with hematoxylin and eosin (H&E) using established protocols. For immunohistochemistry, tissue sections were deparaffinized in xylene and rehydrated with decreasing concentrations of alcohol. Endogenous peroxide was blocked with hydrogen peroxide and antigen retrieval was achieved by using 0.01 mol/L sodium citrate (pH 6.0) for 1 min in a pressure cooker. After blocking in the appropriate normal serum, tissue sections were stained with primary antibodies or with the Mebstain apoptosis kit (TUNEL assay) (code 8445 MBL Woburn, USA). Antibodies used were as follows: rabbit polyclonal anti-mouse/human Ki67 (1∶100; clone ab66155 Abcam Cambridge, UK), mouse anti-mouse/human MelanA (1∶20; clone ab731 Abcam Cambridge, UK). The Vectastain Elite ABC Peroxidase kit (Vector Laboratories, Inc., Burlingame, CA) was used for secondary antibody detection. Visualization was done by using DAB as a substrate (clone ab64238, Abcam Cambridge, UK). A pathologist examined the slides in a blind manner.

### Immunoblotting

For expression of ABCB5 (1∶1000; clone PAB9925, Abnova Taipei City, Taiwan), Nestin (1∶200; clone mab353, Chemicon, Billerica, USA) and CD271/NGFR (1∶200; clone sc-8317, Santa Cruz, USA), cells were grown in 6 well plates. Then cells were released without enzymatic digestion at 4°C. Cell pellets were sonicated for 10 seconds and clarified by centrifugation. Total protein (50 µg) underwent electrophoresis in 7.5% Tris-HCl gels (Bio-Rad, Hercules, CA) and was transferred to polyvinylidene difluoride membranes, blocked and exposed to primary antibody, followed by a secondary antibody conjugated to horseradish peroxidase. Bands were developed using an ECL Plus detection system (Amersham, Piscataway, NJ). Density was quantified using a computer-controlled CCD camera (AlphaImager Imaging Systems, Alpha Innotech, San Legndra, CA).

### Flow Cytometry and Cell Sorting

To detect surface markers on tumor cells, melanoma cell lines were trypsinized or non-enzymatically detached with EDTA and centrifuged at 1500 rpm/min for 5 min. [23] Briefly, Cells were washed with Ca^2+^-free Mg^2+^-free phosphate-buffered saline (PBS) solution, and 4 ml of 1 mM EDTA (Sigma Aldrich) was added to each flask. The flasks were incubated at 37°C for 5 minutes and shaken slowly until cells were detached. Ten milliliters of PBS buffer was then added into each flask and cells picked up to tubes, centrifuged and washed with Ca^2+^/Mg^2+^-free PBS. The number of detached cells was counted with a hemacytometer (Marienfeld, Germany). Cells were then harvested, washed with ice cold 0.5% FBS and 0.02% Sodium Azide in PBS and blocked with a solution of 0.5% FBS and 5 µg/ml purified anti-CD16/CD32 (BioLegend, San Diego, USA) in PBS for 15 min at room temperature. Subsequently, cells were stained for APC conjugated Anti-mouse-CD133 mAb (clone 13A4, eBioscience, San Diego, USA), APC-labeled anti-mouse CD117 (clone 2B8, eBioscience, San Diego, USA), FITC-labeled anti-mouse Sca-1α (clone D7, eBioscience, San Diego, USA), CD271 (clone ab8874, Abcam Cambridge, UK) and Goat polyclonal Secondary Antibody to Rabbit IgG (clone ab6108, Abcam Cambridge, UK) for 30 min on ice. Samples were then washed and analyzed with BD FACSCanto™ II (BD Bioscience, USA).

For the sorting we used MACS® Separation system (#130-090-312, Miltenyl Biotec Inc., USA) according to the manufacturer protocol. Briefly: K1735/M4 cells were stained with APC conjugated anti-mouse-CD133 mAb and cell separation proceeded with anti-APC MicroBeads (#130-090-855, Miltenyl Biotec Inc., USA). The cell partition was done twice with same cells in order to enrich CD133 positive cell population. After cell separation CD133 positive cell population was washed with PBS and prepared for injection (7.5×10^4^ cells/200 µL/mouse) or for verification by FACS.

### Cell Proliferation Assay

Cells were seeded in 96-well plates at a density of 1000 cells per well with DMEM-MSCM. After 24, 48 and 72 hours, cell growth was determined by using the Colorimetric XTT cell proliferation assay (Beit Haemek, Israel). Samples were analyzed with Spectra MR Dynex (Chantilly, USA).

### Soft Agar Assay

Aliquots of 10^4^ melanoma cells were resuspended in 1 ml, 0.35% agar in MSCM. Aliquots were poured into six-well plates on top of a 1.5-ml layer of 0.5% agar in MSCM, allowed to solidify and incubated for 21 days at 37°C in the presence of 5% CO2. The dishes were photographed with a stereoscopic imaging system (SteREO Lumar.V12. Zeiss, Germany) and the number colonies per 1/cm^2^ was estimated after acquisition using ImagJ (NIH, Bethesda, MD) image analysis software.

### Assessment of Tumor Spheroid Formation and Self-renewal Analysis

A 2 ml lower agar layer was prepared by re-suspending 0.5% agar with MSCM, which was then allowed to solidify in 6 well plates. Aliquots of 3×10^4^ melanoma cells in MSCM were plated in suspension conditions on soft agar layer and incubated for 2–16 days at 37°C, in the presence of 5% CO_2_ before analysis.

Sphere Limiting Dilution Analysis (SLDA) was performed as previously described. [24] Briefly, spheres were harvested after 14 days kept in suspension conditions in 6 wells plates, separated with trypsin and plated again in non-adherent conditions in MSCM using dilution aliquots of: 1000, 300, 100, 50, 10 cells/96-wells plate. Fourteen days later, the number of wells without sphere formation was quantified and the data were log transformed and plotted against plating density. A linear regression was used to calculate the frequency of cells capable of proliferating to form a melanoma sphere.

### Quantitative Reverse Transcription-PCR

Total RNA was extracted from mouse melanoma cell lines using an RNeasy Mini Kit (Qiagen, Netherlands), according to the manufacturer’s instructions. Purified RNA was quantified using a NanoDrop® ND-1000 spectrophotometer (NanoDrop Technologies Inc., USA). cDNA was synthesized from 200 ng of total RNA, using the VersoTM cDNA Kit (Thermo Scientific, Epsom, UK) and random hexamers. cDNA PCR amplification was carried out using the Platinum® SYBR® Green qPCR SuperMix-UDG with ROX (Invitrogen Corporation, Grand Island, NY USA) on a Step One Plus Real Time PCR system (Applied Biosystems) with gene-specific oligonucleotide pairs (Sigma Aldrich, Israel). Every gene was checked by three various pairs of primers, listed in Table S1. To ensure the specificity of the reaction conditions, at the end of the individual runs, the melting temperature (Tm) of the amplified products was measured to confirm its homogeneity. Cycling conditions were as follows: 50°C for 2 minutes, 95°C for 2 minutes, 95°C for 15 seconds and 60°C for 30 seconds for a total of 40 cycles. Each sample was analyzed in triplicate. For quantification, standard curves were obtained using serially diluted cDNA amplified in the same real-time PCR run. Results were normalized to ACTB and 18S mRNA levels. The 2-ΔΔCT method was applied to analyze the relative changes in the gene expression. After the quantification procedure, the products were resolved by 2.5% agarose gel electrophoresis to confirm that the reaction had amplified DNA fragments of expected size.

### Statistical Analysis

Student *t* tests or analyses between groups (ANOVA) were used for statistical analysis as appropriate. Differences were considered significant at *P*<0.05. All data is represented as mean ± SD, unless otherwise indicated. All experiments were repeated in triplicate. Data from representative experiments are shown. *In vivo* experiments consisted of 10–15 mice in each experimental group.

## Results

**Figure 1:**
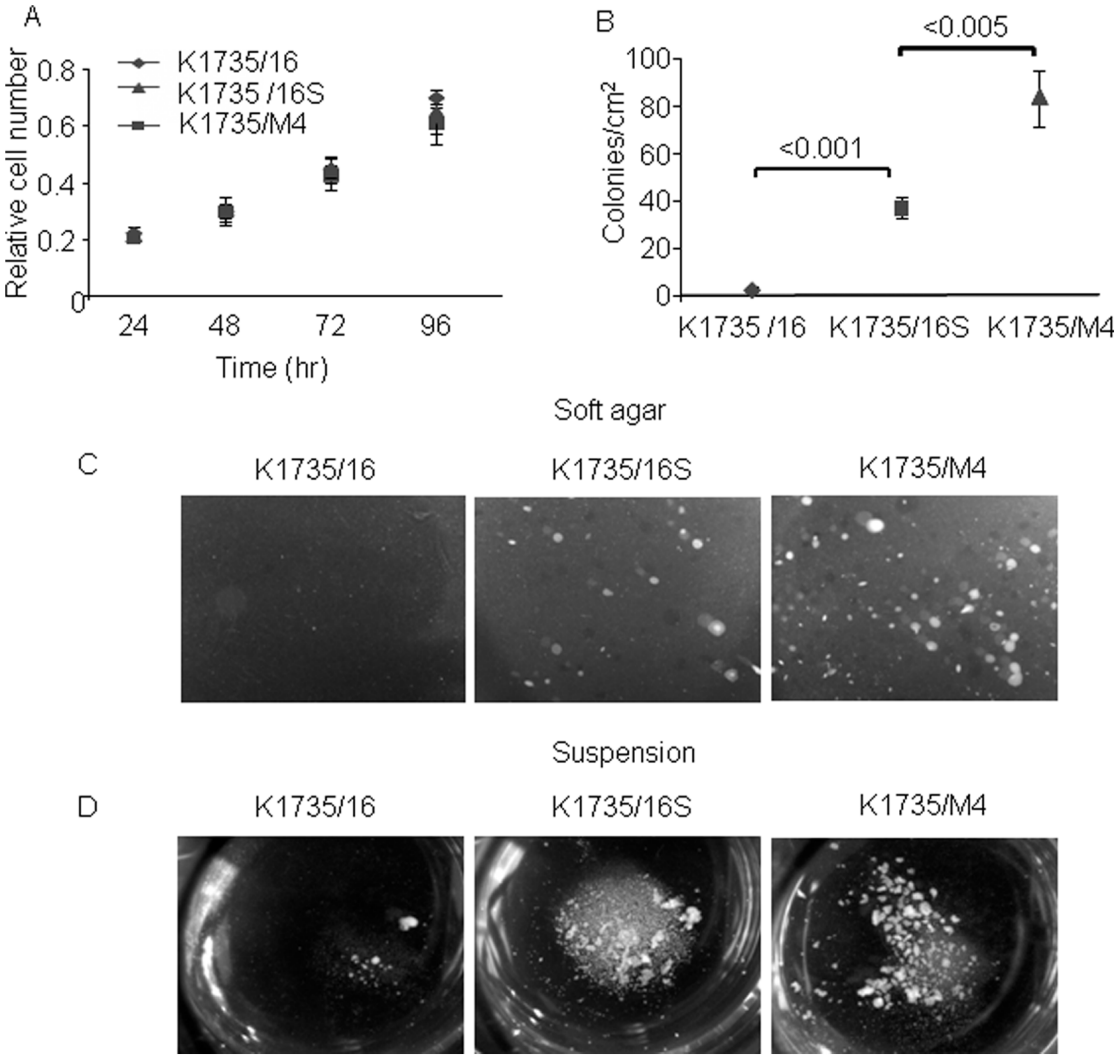
Colonogenic potential of K1735/16, K1735/16S and K1735/M4 melanoma cell lines in soft agar and in non-adherent conditions. (A) Proliferation rate in culture-treated plates was determined after 96 hours by using the XTT assay. Data are the average ± SD of at least three independent experiments performed in triplicate. (B) Mean number of colonies grown on soft agar after 21 days in culture. Data are the average ± SD of five high power fields (P<0.001). (C) Representative microscopic images of tumor spheroids 21 days after plating. (D) Representative microscopic images of tumor spheroids, 6 days after plating in non-adherent conditions.

In order to assess the correlation between cancer cell self-renewal potential and cell fate, we selected murine melanoma cells, which were derived from the same parental cell line, but which have different phenotypes *in-vivo* and *in-vitro*. The K1735 murine melanoma cell line has been used extensively to study melanoma [25]. This cell line, which has a low propensity to form metastases, was induced in C3H/HeN mice by chronic exposure to ultraviolet B radiation. For our study, we choose three cell lines, which were derived from this parental cell line, but which differed significantly in their tumorigenic potential, sphere-forming ability and clonogenicity [26], [27], [28]. The K1735/16 is a non-metastatic cell line and the K1735/M4 cell line was derived from a lung metastasis and has a high metastatic potential. A third cell line, K1735/16S, was derived from the K1735/16 cell line by maintaining the cells in suspension non-adherent conditions for 16 days. All three cell lines exhibited similar proliferation kinetics when grown in culture plates (**Fig. 1A**).

### Soft Agar Colony and Spheroid Formation Potential

Tumor cells grown in three-dimensional multicellular spheroids are considered by many a reliable *in-vitro* model that replicates some of the complex features of solid tumors [29]. We first sought to characterize the clonogenicity and sphere-forming ability of three cell lines K1735/16, K1735/16S and K1735/M4 in the soft agar. The K1735/16 cell line grown in the soft agar failed to form colonies after 21 days in the culture (**Fig. 1B and C**). On the other hand, both the metastatic K1735/M4 cell line and the K1735/16S cell line had prominent a colony formation capacity (**Fig. 1B and 1C**). We next examined whether the same phenotypic heterogeneity is sustained in suspension non-adherent conditions, which is viewed by many to be the closest approximation of *in-vivo* tumorigenicity among the in-vitro assays [30], [31], [32], [33], [34]. The sphere formation capacity was evaluated 6 days after plating with MSCM. A shown in **figure 1D**, the K1735/M4 and K1735/16S cell lines showed a prominent sphere-forming ability, whereas the K1735/16 cell line failed to grow in these stress conditions. However, when K1735/16 cells were kept at these conditions for 16–20 days, a few spheroids could be detected in some of the plates.

### Stemness Potential

**Figure 2:**
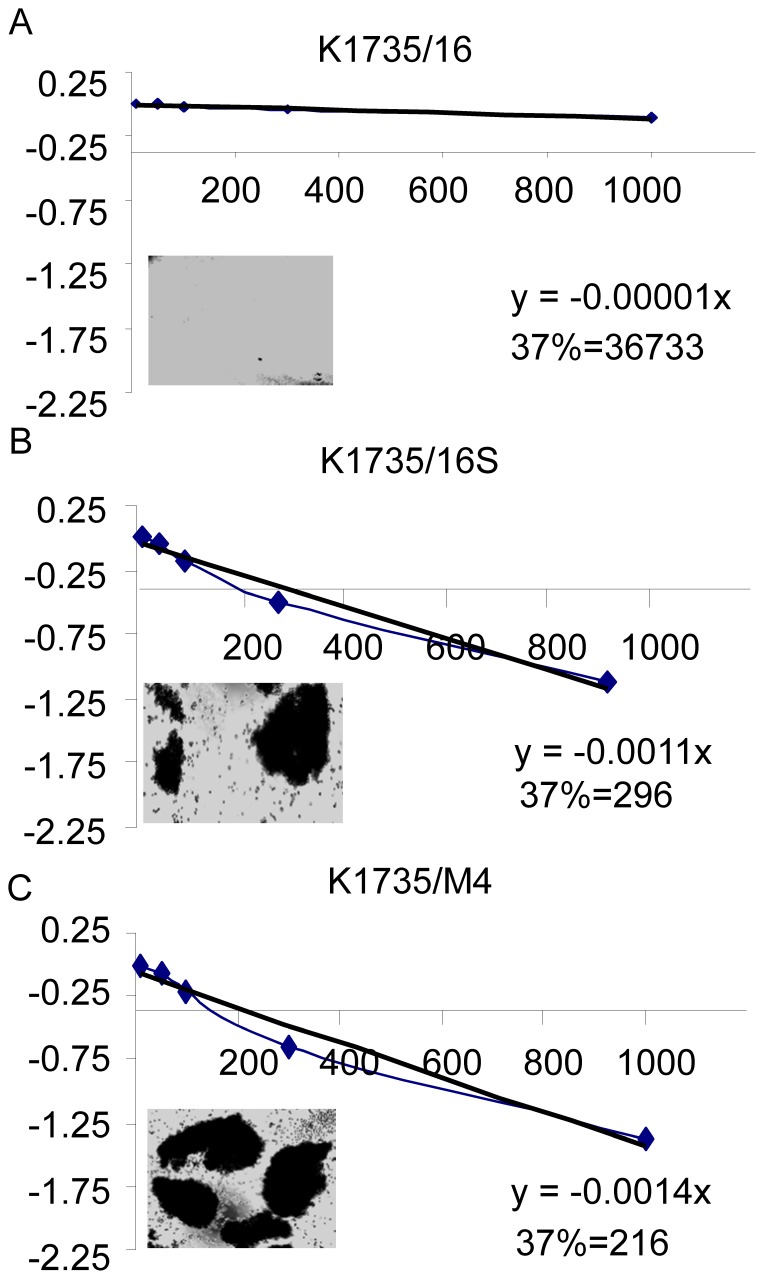
Self-renewal potential of melanoma cells in non-adherent conditions. SLDA performed after the second reseeding with MSCM revealed that the frequency of cells capable of self renewing was A. 1/216 for the K1735/M4 cell line, B. 1/296 for the K1735/16S cell line and C. 1/74720 for the K1735-16 cell line. The intercept of log (37% negative wells) was used to calculate the sphere-forming frequency (see materials and methods).

To evaluate the self-renewal potential of the three cell lines, we carried out a series of dilution assays by using of SLDA in serum-free media, directed to examine the ability of a limiting dilution analysis of melanoma cells to form spheroids in non-adherent conditions and established a frequency analysis of melanoma sphere formation. [24], [35], [36] All three cell lines had a similar proliferation potential when plated on 96-wells culture plates in normal conditions (**Fig. 2A**). In the contrast, the self-renewal potential in serum-free media of the non-metastatic cell line, K1735/16, differed significantly from the other two cell lines. SLDA performed after the second reseeding revealed that the frequency of cells capable of self renewing was 1/216 for the K1735/M4 cell line, 1/296 for the K1735/16S cell line and 1/36733 for the K1735-16 cell line with MSCM (Fig. 2). In addition, K1735-16 cells were not capable to form spheres in adeherent conditions, whereas K1735/M4 cells spontaneously formed sphares in serum-free media.

Taken together, these data suggest a phenotypic heterogeneity of single murine melanoma cells, which were derived from the same parental cell line. The self-renewal potential in the suspension non-adherent assay is comparable to the sphere-forming ability and clonogenicity in the soft agar assay.

### Tumorigenic Potential in SCID Mice

**Figure 3:**
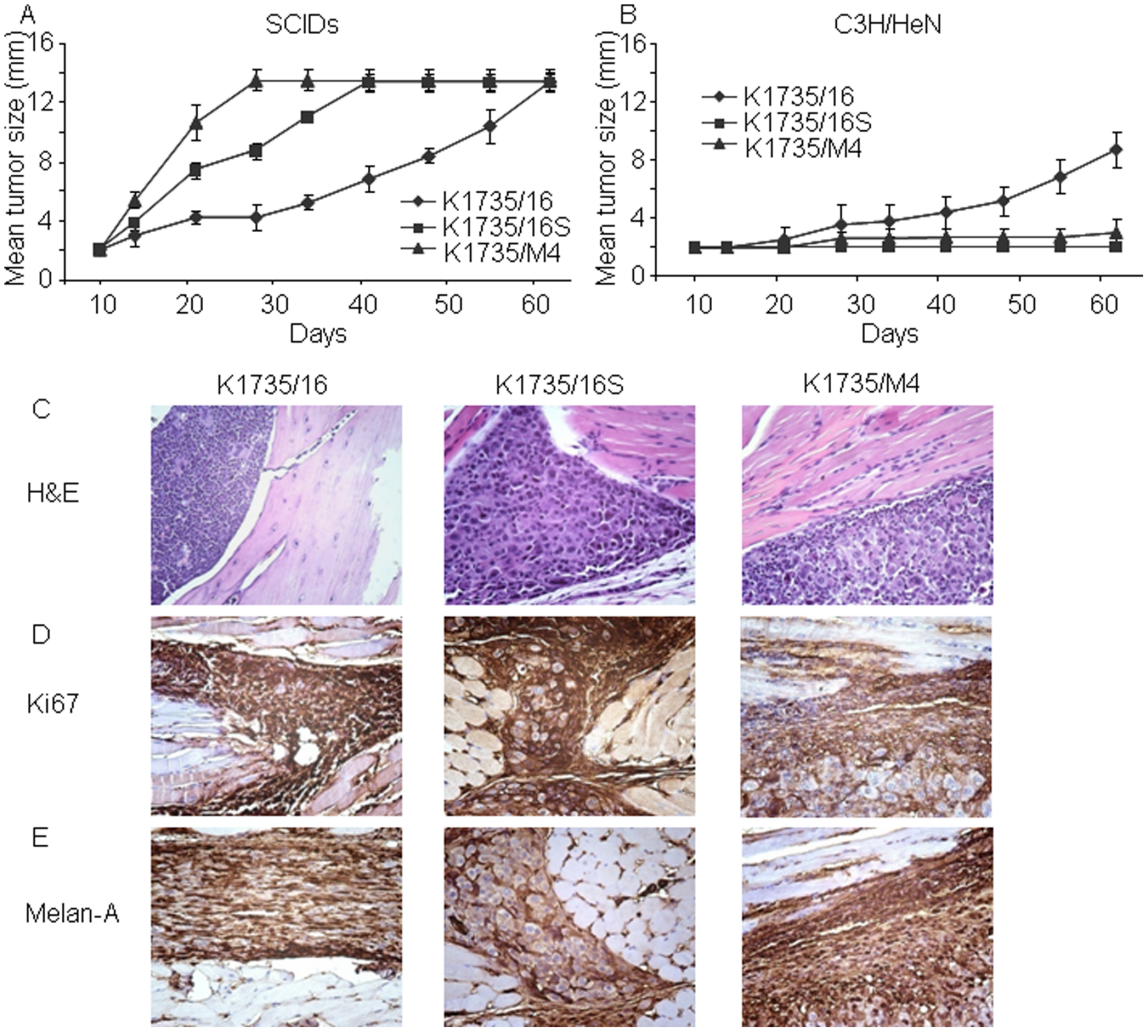
Tumorigenic potential and immunohistological characterization of murine melanoma cell lines in SCID and syngeneic mice using intra footpad injections. (A) Melanoma cell lines (2×10^5^) injected to the footpad of SCID mice. The K1735/M4 cell line showed the highest tumor growth kinetics, followed by the K1735/16S cell line. The K1735/16 cell line had the slowest tumor growth (n = 5–6 per group, P<0.001). (B) The same concentration of cells injected into the footpad of syngeneic C3H/HeN mice. This time, the K1735/M4 and K1735/16S showed minimal tumorigenic potential, whereas the K1735/16S cell line had the highest tumor growth kinetc (experiments performed 3 consecutive times with n = 10–15 animals per group; P<0.001 between the K1735/16 and the K1735/M4 or the K1735/16S cell lines). (C) Representative histologic features of tumors grown in immune-competent animals, 16 days after implantation. (D) Immunohistochemical analysis with anti-Ki-67 Ab (a proliferation marker) showing similar expression in the three cell lines. (E) Immunohistochemical staining with anti-Melan A Ab (a melanoma marker) showed positive expression in all tumors but not in adjacent normal tissue.

Next we sought to evaluate the tumorigenic potential of the three cell lines in immunocompromised mice using orthotopic intra footpad injections. This was assessed by injecting 2×10^5^ freshly dissociated K1735/16, K1735/M4 and K1735/16S cancer cells into the footpad of SCID mice. Palpable tumors were evident in all mice within 20 days. As shown in **Figure 3A**, the metastatic cell line, K1735/M4, exhibited the highest growth kinetics, followed by the K1735/16S cell line, whereas the non-metastatic/non-colonogenic 1735/16 cell line had the lowest tumor growing potential (n = 6–7 mice per group, *P*<0.001). These data suggest that the self-renewal potential *in-vitro* can predict the *in-vivo* tumorigenic potential in SCID mice.

### Tumorigenic Potential in Immune-competent Mice

Next, we wanted to evaluate whether the tumorigenic potential of the three cell lines, as revealed by the two *in-vivo* and *in-vitro* assays described above, can predict the actual fate of cancer cells in immune-competent animals. This question can only be addressed in syngeneic mouse models that can reflect the anti-tumor immune reaction and the microenvironment response. Therefore, we repeated the intra footpad injections previously performed in SCID mice, but this time using syngeneic C3H/HeN mice.

**Figure 4:**
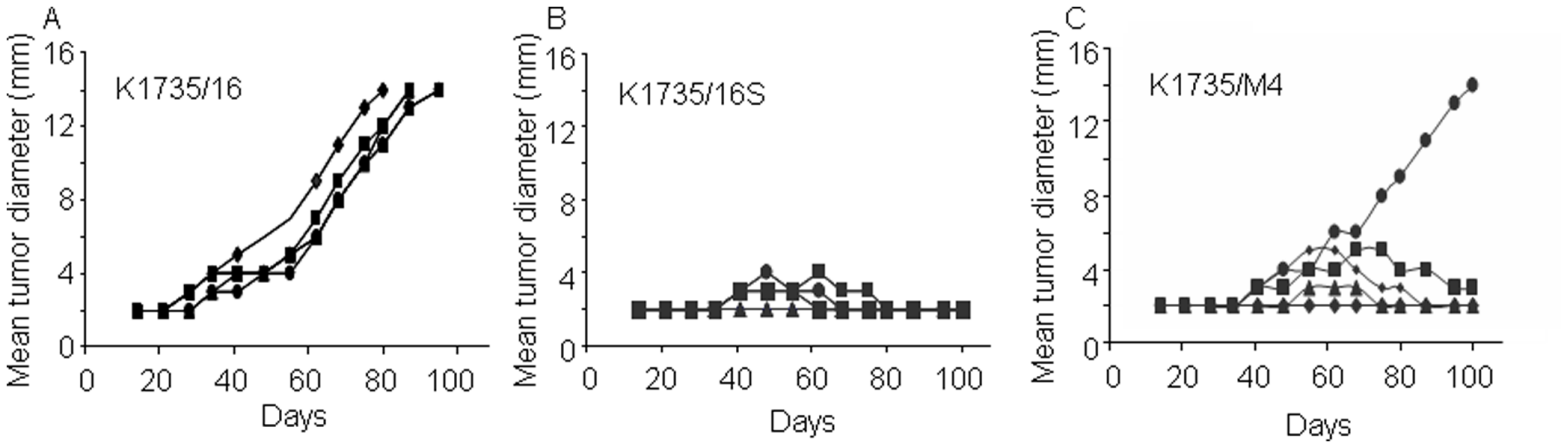
Spontaneous melanoma tumor regression in syngeneic mice. (A–C) Graphs show tumor growth curves of all three cell lines in syngeneic mice, 1–100 days after injection of 2×10^5^ cells. Each graph shows tumor development in individual animal (n = 5 per group).

To our surprise, the fate of the cancer cells in the syngeneic model was quite the opposite than that previously described in SCID mice. As shown in **Figure 3B**, K1735/16 cells injected into the intra footpad of C3H/HeN mice showed the highest cancer growth kinetics, whereas only small numbers of animals developed tumors after injection of the K1735/M4 or K1735/16S cell line (experiments performed 3 times with n = 10–15 mice per group, *P*<0.001). Histologic analysis of tumors with H&E, and immunohistochemical staining with anti-Ki67 Ab (a proliferation marker) or anti-Melan-A Ab (a melanoma marker) revealed similar expression of these proteins by the three cancer cell line-derived tumors (**Fig. 3D–E**). Further analyses of single tumors revealed that all mice injected to the footpad of syngeneic C3H/HeN with the K1735/16 cell line exhibited macroscopic tumors 30–100 days after injection (**Fig. 4A**). In contrast, some mice injected with the K1735/M4 or K1735/16S cell lines actually developed small tumors 40 days after injection, but these tumors regressed spontaneously in 90–100% of mice within 80 days after the experiment was initiated (**Fig. 4B and C**).

**Figure 5:**
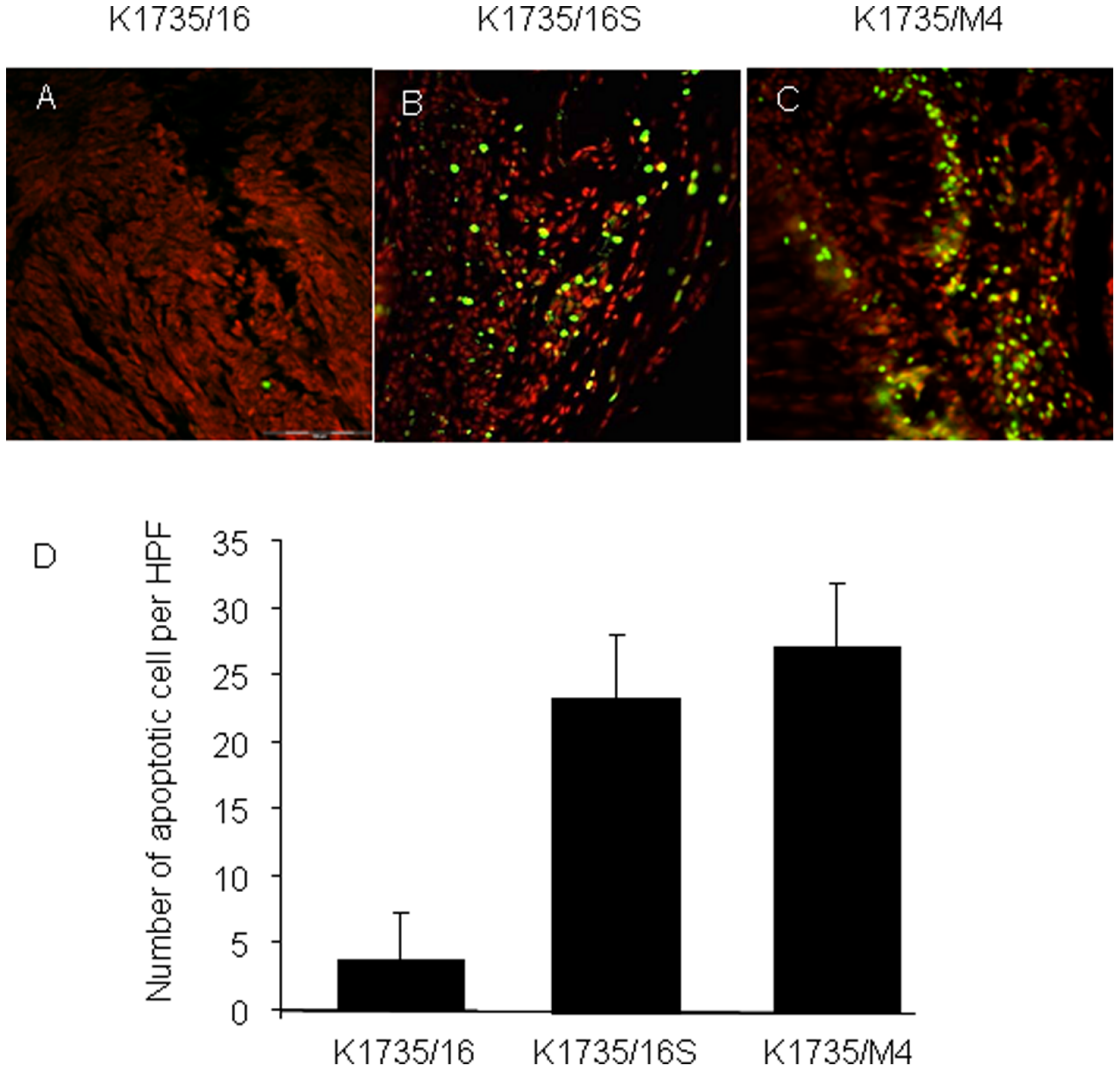
Apoptosis rate of murine melanoma cells in a syngeneic intra footpad model. Apoptosis was assessed using the TUNEL assay. (A–C) Representative microscopic pictures (magnification X20) of FITC-positive apoptotic tumor cells (green) and counter staining with propidium iodide (red). (D) Percentage of apoptotic cells was quantified by the mean number of FITC-positive cells in 6 microscopic high power fields. Data are expressed as the mean+SD (n = 6 tumors, P<0.001). TUNEL analysis revealed that the number of apoptotic cells in K1735/16 cell line-derived tumors was significantly lower than in K1735/16S and K1735/M4 cells lines-derived tumors.

Spontaneous tumor regression in the K1735/M4 and K1735/16S cell lines can be due to cells undergoing apoptosis days after implantation or cells remaining dormant for a long period of time. We therefore examined the number of apoptotic cells in these tumors 40 days after injection into footpads of C3H/HeN mice. TUNEL analysis of apoptosis revealed significantly higher numbers of apoptotic cells in K1735/16S and K1735/M4 cell line-derived tumors, compared to that in K1735/16-derived tumors (**Fig. 5**). This finding indicates that the tumorigenic potential of melanoma is assay specific and that the *in-vivo* phenotypic heterogeneity of different subpopulations of cells in normal animals cannot be predicted solely by its stemness potential.

### Expression Profile of Stem Cell Markers

**Figure 6:**
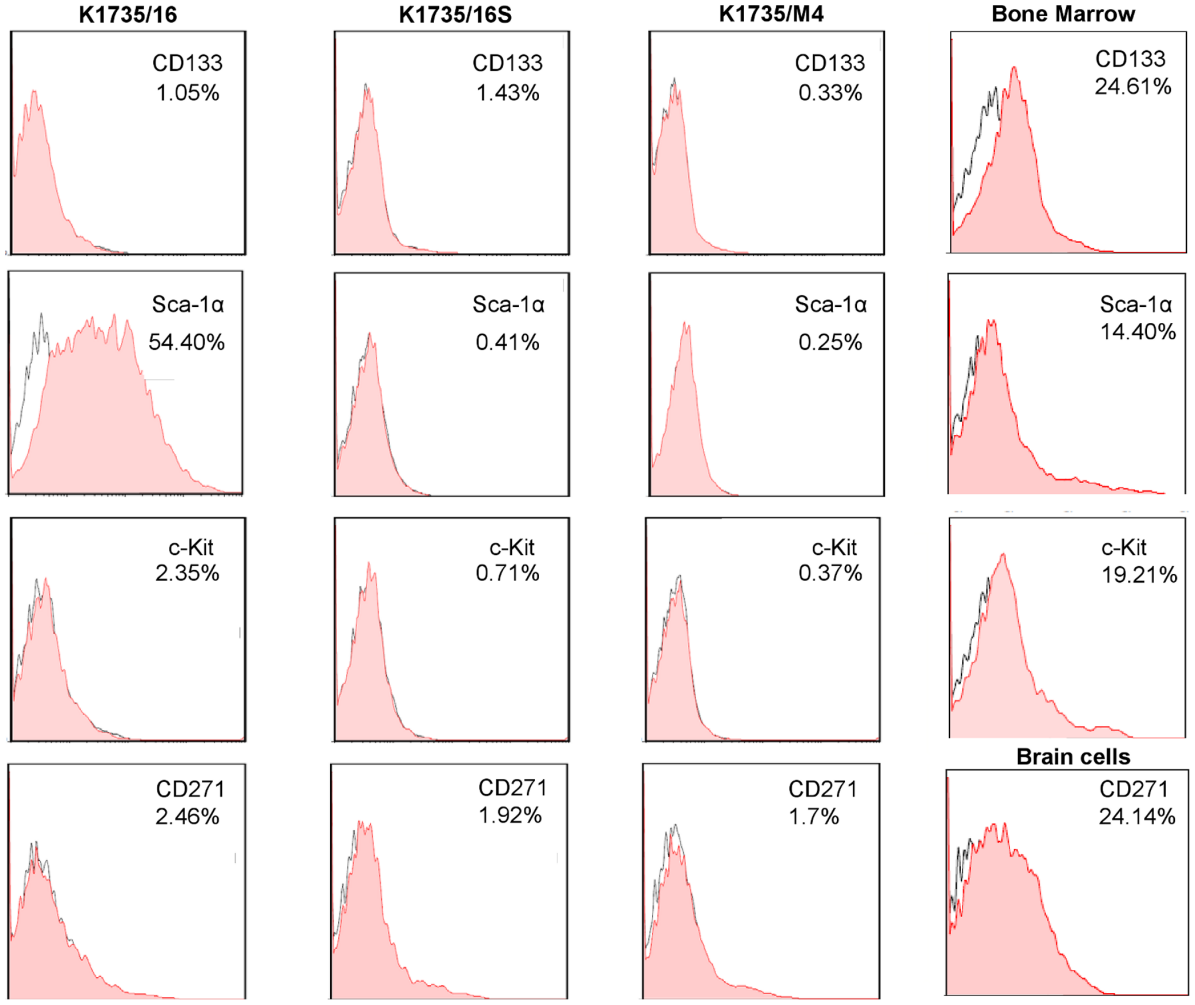
Stem cell marker expression by K1735/16, K1735/M4 and K1735/16S melanoma cell lines measured by FACS. Expression of stem cell markers was determined using anti- Sca-1α, c-Kit, CD133 and CD271 Abs. As opposed to the other markers, SCA-1α was expressed in >50% of the cells in the K1735/16 cell line, but not in the other cell lines. Results are from one of three representative experiments. The percentage of positive cells and markers are shown in the upper right corner. B16 mouse melanoma cells, bone marrow (BM) or brain cells (BC) were used as positive controls.

Evidence suggests that tumorigenic cells can be distinguished from non-tumorigenic cells based on expression of specific markers. Therefore we examined the ability of reported stem cell markers to predict the clinical phenotype of the three melanoma cell lines. It was previously shown that c-Kit, CD133 and CD271 expression assays can distinguish tumorigenic from non-tumorigenic melanoma cells [13], [37], [38], [39]. We also investigated the expression of Sca-1α, that was shown to be associated with tumorigenic potential in breast, prostate and lung cancers [13], [37], [38], [39]. We therefore examined the expression of these markers by flow cytometry in all three cell lines. Cells were detached using trypsin cleavage or in enzymatic-free conditions using EDTA. **Figure 6**-trypsin treated cells and **Figure S1**-EDTA treated cells shows that c-Kit, CD133 and CD271 positive cells were detected only in the minority of cells. In contrast, Sca-1α expression was found in >50% of the K1735/16 cells, but not in the other cell lines. These results were confirmed by quantitative RT-PCR analyses.

**Figure 7:**
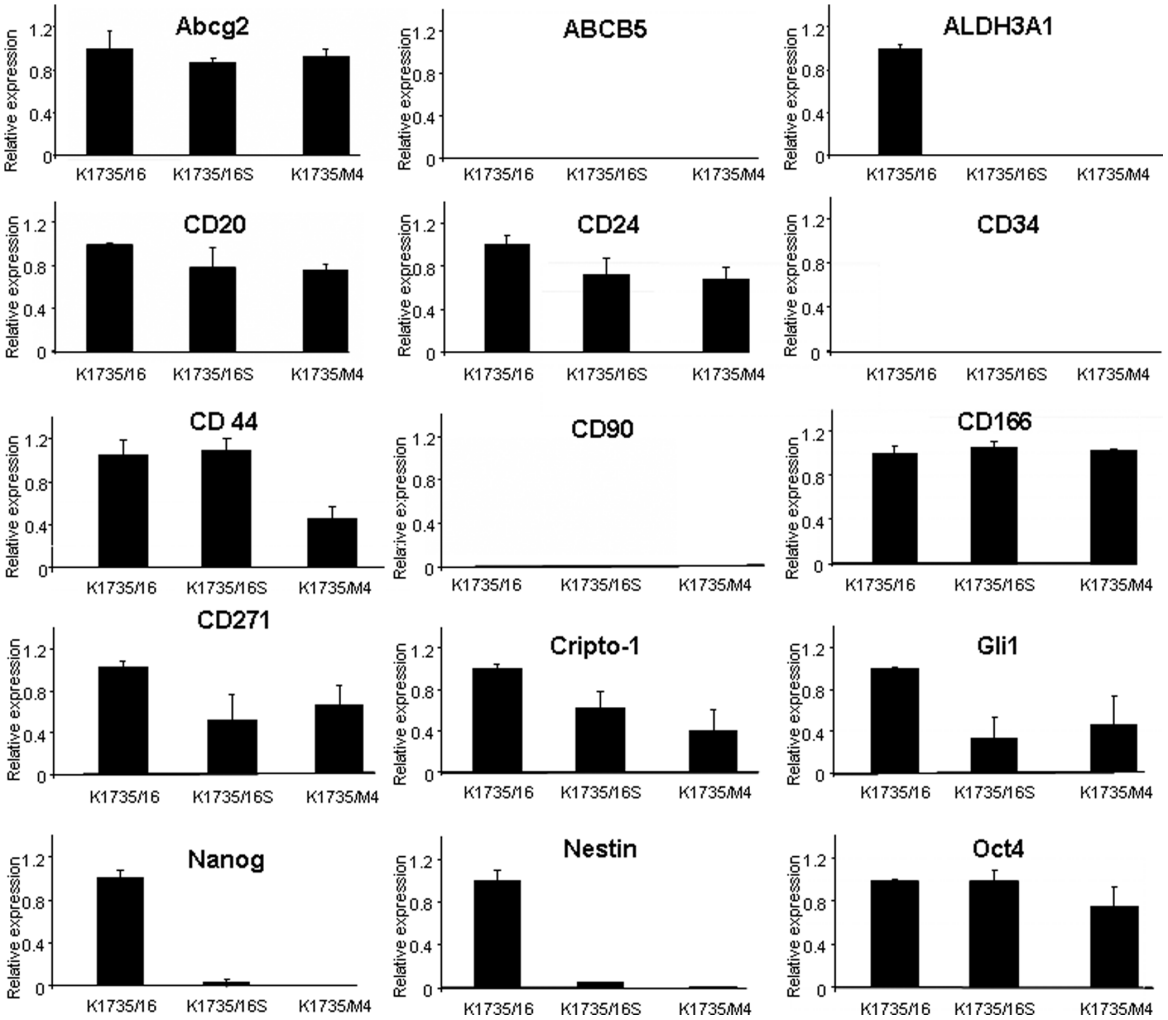
Stem cell marker expression by the three melanoma cell lines determined by quantitative RT-PCR analysis. Relative expression of 15 markers previously suggested for use to predict the self-renewal potential of cancer cells. ALDH3A1, Nanog and Nestin are expressed mostly in the K1735/16 cell line, but not in the K1735/16S and K1735/M4 cell lines. Other stem cell markers including SOX2 did not show any differential expression between the three cell lines. Data are the average ± SD from three different experiments. Freshly dissociated bone marrow and brain cells were used as positive controls (right column).

**Figure 8:**
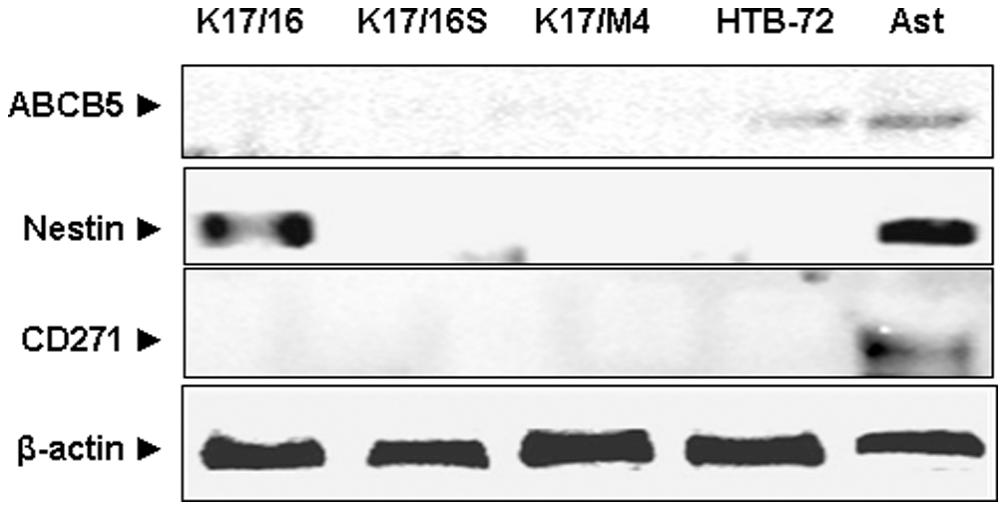
Western blot analysis of ABCB5, Nestin and CD271 expression by mouse melanoma cell lines. These markers were previously suggested for use to predict the melanoma self-renewal potential. HTB-72 human melanoma cell line and murine astrocytes (Ast) were used as positive controls. Note that the only marker that was expressed by the murine melanoma cell lines was Nestine, which was expressed in the non-metastatic cell line (K1735/16).

We further sought to identify other CSC markers expressed by melanoma cells which can predict their tumorigenic phenotype. Thus, we screened another 16 markers previously shown to be expressed in CSCs [12], [13], [21], [40], [41], [42], [43], [44], [45], [46], [47]. Using quantitative RT-PCR analysis we were unable to detect higher expression of Abcg2, ABCB5, CD20, CD24, CD34, CD44, CD166, Oct4, Cripto-1, CD-90, Gli1 or Sox2 in any of the K1735/M4, K1735/16S and K1735/16 cell lines (**Fig. 7**). We confirmed these results in some of the markers by Western blot (**Fig. 8**). Interestingly, three markers Nanog, ALDH3A1 and Nestin, were expressed in the K1735/16 cell line, but not in its daughter cell line K1735/16S or in the metastatic K1735/M4 cell line. In total, we screened 19 different markers that were previously suggested to be associated with the tumorigenic potential of cancer cells, and in all of them, the highly-tumorigenic cell lines K1735/M4 and K1735/16S had similar or lower expression compared to the low-tumorigenic cell line.

Finally, we sought to confirm our results by clonal cell sorting at the single cell level. This assay was directed to determine whether sub populations of K1735/M4 can give rise to enhanced clonogenicity and self-renewal. We focused on CD133, a CSC marker that was repeatedly used by multiple investigators to examine hierarchical organization, symmetric and asymmetric cell division of melanoma stem cells. [41], [48] We have used MACS and FACS technology to purify CD133(+) cellular subsets from the K1735/M4 metastatic cell line. Cells (75×10^4^ cells per mouse) were injected to the footpad of syngeneic mice (n = 5–6 mice per group). **Figure S2** shows that regardless of its clonal feature, CD133(+) K1735/M4 cells did not induce footpad tumors >45 days after injection.

## Discussion

The self-renewal potential of a cancer cell can be estimated under a particular assay condition. Subcutaneous xenotransplantation of cancer cells in SCID mice is a well-established assay used to estimate the tumorigenic potential of these cells. However, highly immunocompromised animal models cannot address the question of the actual fate of these cancer cells [1]. The clinical phenotype of tumor cells may be regulated by paracrine signals derived from the immune system. For example, a more immunogenic subpopulation of cells with high tumorigenic potential may remain dormant for many years or undergo apoptosis under regulation of an active immune system [49]. In contrast, cells with low self-renewal potential, may escape the immune system and form tumors, if their immunogenic capacity is low. Hence, the questions of tumorigenic potential and the cancer cell’s fate can be only determined using assays that approximate the active immune response of normal subjects and in an orthotopic environment.

In this paper, we sought to determine the tumorigenic potential and the cancer cell’s fate in a murine intra footpad melanoma model. We examined three cell lines that were derived from the same parental cell line. This included a non-metastatic cell line (K1735/16), a metastatic cell line (K1735/M4) and a cell line (K1735/16S), which was selected after growing the K1735/16 cell line in suspension non-adherent conditions. The K1735/16 cell line had low self-renewal potential *in-vitro,* while the other cell lines showed high self-renewal potential in non-adherent conditions. As expected, the self-renewal capacity of the K1735/M4 and K1735/16S cell lines correlated with their tumorigenic potential seen in SCID mice. Once the self-renewal potential and tumorigenicity were identified, we evaluated which of these cells produced tumors in an orthotopic/syngeneic melanoma model. Our analysis of tumor growth in immune-competent mice revealed that cell lines, which exhibited a high-tumorigenic potential, underwent spontaneous regression, while the low-tumorigenic cell line developed in all mice tested. These data indicate that in the tested syngeneic model, the self-renewal potential could not predict the actual ability of cells to form tumors in normal animals. The most probable explanation for this finding is that the immunogenic traits of these cells govern their actual tendency to produce tumors. Indeed, in the K1735/M4 and K1735/16S cell lines, we were able to detect high apoptosis levels, compared to the K1735/16 cell line.

Previous studies with melanoma suggested that a small subpopulation of cells that can be distinguished based on specific markers are responsible for tumor formation [12], [39]. We have not been able to identify any marker that predicts the self-renewal potential of the tested cell lines, despite examining 19 markers related to CSCs [50]. In this regard, our data is similar to results from primary mouse melanoma studies, which reported tumorigenic activity in a high percentage of single cells, which did not concur with the CSC model [42]. It is therefore possible that the melanoma cancer cells tested here have a stochastic probability of forming tumors and that genetic or epigenetic modification of various clones differentially contributes to tumorigenic potential or immunogenic phenotype and hence to cell fate.

Previous studies suggested that tumorigenic cells could be distinguished from non-tumorigenic ones by reduced immunogenicity, allowing them to proliferate more extensively by escaping immune detection [51]. The models we have examined here raised another possibility, that cells with high-tumorigenic activity may be more immunogenic and hence are more susceptible to immune-regulation.

## Supporting Information

Figure S1 Expression of stem cell markers by K1735/16, K1735/M4 and K1735/16S melanoma cell lines after 1 mMol EDTA detachment measured by FACS. Melanoma cell lines were detached by 1 mMol EDTA. Expression of stem cell markers was determined using anti- Sca-1α, c-Kit, CD133 and CD271 Abs. The percentage of positive cells and markers are shown in the upper right corner. Results are from one of two representative experiments.
(TIF)

Figure S2 K1735/M4 CD133+ melanoma cells purified by MACS technology and injected to footpad of syngeneic C3H/HeN mice. (A) FACS analysis of pre and post sorted K1735/M4 CD133+ melanoma cells. Results are from one of two representative experiments. (B) Melanoma cell lines K1735/16, K1735/M4 and sorted K1735/M4 CD133+ (7.5×10^4^) were injected intra footpad of syngeneic C3H/HeN mice (n = 5–6 per group, P<0.001).
(TIF)

Table S1 Primer pairs used for real time PCR.
(DOCX)
