# Supplementary figures and images for: Association between Tumorigenic Potential and the Fate of Cancer Cells in a Syngeneic Melanoma Model
Source: PLoS One. 2013 Apr 23;8(4):e62124. doi: 10.1371/journal.pone.0062124 (PMC3633909; doi:10.1371/journal.pone.0062124)

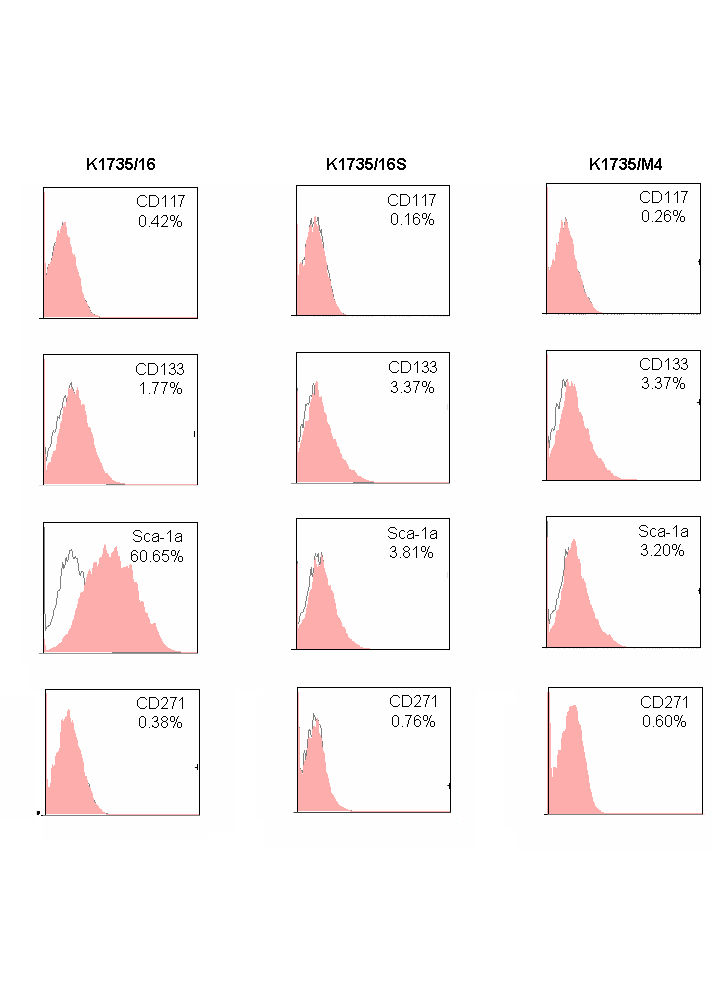

Supplement: Figure S1 — Expression of stem cell markers by K1735/16, K1735/M4 and K1735/16S melanoma cell lines after 1 mMol EDTA detachment measured by FACS. Melanoma cell lines were detached by 1 mMol EDTA. Expression of stem cell markers was determined using anti- Sca-1α, c-Kit, CD133 and CD271 Abs. The percentage of positive cells and markers are shown in the upper right corner. Results are from one of two representative experiments. (TIF) [file pone.0062124.s001.tif]

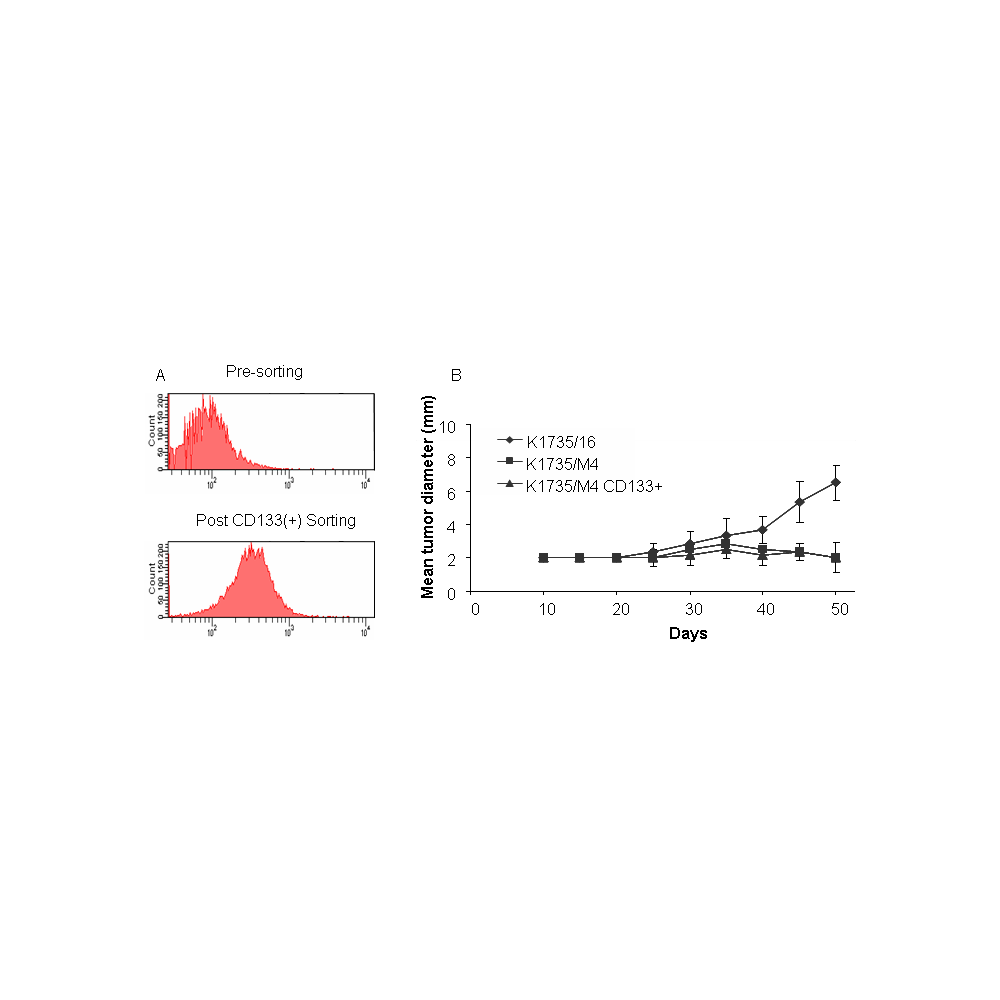

Supplement: Figure S2 — K1735/M4 CD133+ melanoma cells purified by MACS technology and injected to footpad of syngeneic C3H/HeN mice. (A) FACS analysis of pre and post sorted K1735/M4 CD133+ melanoma cells. Results are from one of two representative experiments. (B) Melanoma cell lines K1735/16, K1735/M4 and sorted K1735/M4 CD133+ (7.5×10^4^) were injected intra footpad of syngeneic C3H/HeN mice (n = 5–6 per group, P<0.001). (TIF) [file pone.0062124.s002.tif]
