# Supplementary material for: Association between Tumorigenic Potential and the Fate of Cancer Cells in a Syngeneic Melanoma Model
Source: PLoS One. 2013 Apr 23;8(4):e62124. doi: 10.1371/journal.pone.0062124 (PMC3633909; doi:10.1371/journal.pone.0062124)
Supplement: Table S1 — Primer pairs used for real time PCR. (DOCX) [file pone.0062124.s003.docx]

| **Name** | **Sequence** | **Product size** |
| --- | --- | --- |
| **1)Abcg2 F** | AGCAGCAAGGAAAGATCCAA | **117bp** |
| **1)Abcg2 R** | CCCATCACAACGTCATCTTG |  |
| **2)Abcg2 F** | TGGTTTGGACTCAAGCACAG | **103bp** |
| **2)Abcg2 R** | GGAATACCGAGGCTGATGAA |  |
| **3)Abcg2 F** | TGAGGCCTGACAGTTCTCCT | **116bp** |
| **3)Abcg2 R** | TTGTTTCTCTGCGACATTGG |  |
| **1)ABCB5 F** | GTGGCTGAAGAAGCCTTGTC | **100bp** |
| **1)ABCB5 R** | CCTTTGCGTCCTTTAAGTGC |  |
| **2)ABCB5 F** | CCGGAAGGCATGTTCATAGT | **101bp** |
| **2)ABCB5 R** | CCAGCTTTGGCTTTGGAATA |  |
| **3)ABCB5 F** | AAAGCCATGCTCTACCAGGA | **111bp** |
| **3)ABCB5 R** | AGTTGCTGCACCCTGAATCT |  |
| **1)ALDH3A1 F** | CCCCTGGCACTCTATGTGTT | **116bp** |
| **1)ALDH3A1 R** | GTGGGCACAGTGATGTGAAC |  |
| **2)ALDH3A1 F** | TCACATCACTGTGCCCACTT | **120bp** |
| **2)ALDH3A1 R** | AGAGACCTCACCAGGCAAGA |  |
| **3)ALDH3A1 F** | TATGGGAGGATCATCAACGAC | **114bp** |
| **3)ALDH3A1 R** | GGTGGGAGCTATGTATCGTGA |  |
| **1)CD20 F** | ATCCTTGCCAGTGGAAAATG | **106bp** |
| **1)CD20 R** | GGCCACAAGAGATGAGGGTA |  |
| **2)CD20 F** | TGATCTCTGCCTTCTTCCAGA | **104bp** |
| **2)CD20 R** | AGCTGACAGCAGAACCACATT |  |
| **3)CD20 F** | TTCAAACTTCCAAGCCGTATG | **120bp** |
| **3)CD20 R** | ACAGAATGCCCAAGAACACAG |  |
| **1)CD24 F** | CTTCTGGCACTGCTCCTACC | **100bp** |
| **1)CD24 R** | TACTTGGATTTGGGGAAGCA |  |
| **2)CD24 F** | CAGGCCAGGAAACGTCTCTA | **100bp** |
| **2)CD24 R** | CAATTCGAGGTGGACCTGTT |  |
| **2)CD24 F** | TCTTTTGTTCGCATGGTCAC | **100bp** |
| **2)CD24 R** | TCCTTCTTCCCAGGAAGGTT |  |
| **1)CD34 F** | GACAACATGTGGTGGCTGAC | **107bp** |
| **1)CD34 R** | AGCTGAAGGCAGCATGAAGT |  |
| **2)CD34 F** | GCACCACTGGTTATTTCCTGA | **105bp** |
| **2)CD34 R** | CATGTGCAGCCTCTTTTCTTC |  |
| **3)CD34 F** | TAAGACCACACCAGCCATCTC | **107bp** |
| **3)CD34 R** | GGGAAGTCTGTGGTTGTGAAA |  |
| **1)CD44 F** | GTGGGCAGAAGAAAAAGCTG | **118bp** |
| **1)CD44 R** | TTGTTCACCAAATGCACCAT |  |
| **2)CD44 F** | CCCTACCCCAAGTGAAGACTC | **112bp** |
| **2)CD44 R** | TGTCCAGGAAACATCCTCTTG |  |
| **3)CD44 F** | TGAGGGCACAAGAAGAGTCAT | **109bp** |
| **3)CD44 R** | ACCACTATGGCAAGCAATGTC |  |
| **1)CD90 F** | CGCTCTCCTGCTCTCAGTCT | **109bp** |
| **1)CD90 R** | GTTATTCTCATGGCGGCAGT |  |
| **2)CD90 F** | GTCTTGCAACCTGCCTCTTC | **107bp** |
| **2)CD90 R** | GGAGAGGATCCTTGGGAAAG |  |
| **3)CD90 F** | CTCCAACCAGCCCTATATCAAG | **116bp** |
| **3)CD90 R** | TTTTATTGGAGCTCATGGGATT |  |
| **1)CD117 F** | ACAAGAGGAGATCCGCAAGA | **100bp** |
| **1)CD117 R** | AGCAAATCATCCAGGTCCAG |  |
| **2)CD117 F** | TCCATGTCAGAGTGGCTTTG | **113bp** |
| **2)CD117 R** | TGTGCTCCCTGCTATGTGAG |  |
| **3)CD117 F** | TTGGATCAGCAAATGTCACAA | **105bp** |
| **3)CD117 R** | CGTTTTCTCCATCGGTTACAA |  |
| **1)CD133 F** | TCAAAGGGACCCAGAAACTG | **118bp** |
| **1)CD133 R** | GCCTTGTTCTTGGTGTTGGT |  |
| **2)CD133 F** | ACGTTTGTTGTTGGTGCAAA | **109bp** |
| **2)CD133 R** | ATTGCCATTGTTCCTTGAGC |  |
| **3)CD133 F** | ACCAACCTGAGCTCTGTGAGA | **110bp** |
| **3)CD133 R** | CTGCTTAGGCTTGGTCTGATG |  |
| **1)CD166 F** | CAGGCCTTGGATGGTACACT | **101bp** |
| **1)CD166 R** | TTTCCATTTGCCAAACATGA |  |
| **2)CD166 F** | TTCAGGAGGTTGAGGGACTG | **101bp** |
| **2)CD166 R** | CAGTCCACTGGGGTCAGTTT |  |
| **3)CD166 F** | AAGAGAGGAGCGGATTGGATA | **115bp** |
| **3)CD166 R** | TTGAACCATGAATTGCTGTCA |  |
| **1)CD271 F** | TACCAGGACGAGGAGACTGG | **114bp** |
| **1)CD271 R** | CTCTGGGCACTCTTCACACA |  |
| **2)CD271 F** | AGCTAGAAGCTGAGCGCTGT | **115bp** |
| **2)CD271 R** | GACACCCCTAGAAGCAGCAG |  |
| **3)CD271 F** | AGCGGCATCTCTGTGGAC | **114bp** |
| **3)CD271 R** | GGTCAGGGGCAGGCTACT |  |
| **1)Cripto-1 F** | TTGGGACCAGAAAGAACCTG | **117bp** |
| **1)Cripto-1 R** | ATGCAAGTCCCTCCATTCAG |  |
| **2)Cripto-1 F** | AACCCCTGTCTCAAAAAGGAA | **117bp** |
| **2)Cripto-1 R** | AATCCACTCTGTTTCCCCAGT |  |
| **3)Cripto-1 F** | TCGAAGATGGGGTACTTCTCAT | **102bp** |
| **3)Cripto-1 R** | ATCTCTGATGGCAAGGTCTCTC |  |
| **1)Gli1 F** | TGTTGTGGGAGGGAAGAAAC | **102bp** |
| **1)Gli1 R** | TGGCAGGGCTCTGACTAACT |  |
| **2)Gli1 F** | TCAATCCAATGACTCCACCA | **118bp** |
| **2)Gli1 R** | CAAAAGGGCAGACCAGAAAG |  |
| **3)Gli1 F** | CCTGAGCCTGAGTCTGTGTATG | **109bp** |
| **3)Gli1 R** | CGTGGATATGCTCACTGTTGAT |  |
| **1)Nanog F** | CCAGGTTCCTTCCTTCTTCC | **106bp** |
| **1)Nanog R** | GCCCTCTTCTGGAGTGTCTG |  |
| **2)Nanog F** | TTTGGTTGTTGCCTAAAACCTT | **115bp** |
| **2)Nanog R** | AGAATTCGATGCTTCCTCAGAA |  |
| **3)Nanog F** | CACTGACATGAGTGTGGGTCTT | **119bp** |
| **3)Nanog R** | AGCAAGAATAGTTCTCGGGATG |  |
| **1)Nestin F** | GGAAGAGAGTGGGGAAGAGG | **116bp** |
| **1)Nestin R** | CATCCTGGACCTTGACACCT |  |
| **2)Nestin F** | GGTCTGGACACAGAAGAAATCC | **101bp** |
| **2)Nestin R** | TTCTGGGTTTTCAGAGACATCA |  |
| **3)Nestin F** | AGGCGCTGGAACAGAGATT | **102bp** |
| **3)Nestin R** | GACCCTGCTTCTCCTGCTC |  |
| **1)Oct4 F** | CACGAGTGGAAAGCAACTCA | **112bp** |
| **1)Oct4 R** | TTCATGTCCTGGGACTCCTC |  |
| **2)Oct4 F** | ATCAGCTTGGGCTAGAGAAGG | **120bp** |
| **2)Oct4 R** | AAGGTGTCCCTGTAGCCTCAT |  |
| **3)Oct4 F** | CTTGCAGCTCAGCCTTAAGAA | **104bp** |
| **3)Oct4 R** | CCGATTTGCATATCTCCTGAA |  |
| **1)Sca-1**α **F** | CCATCAATTACCTGCCCCTA | **112bp** |
| **1)Sca-1**α **R** | GGCAGATGGGTAAGCAAAGA |  |
| **2)Sca-1**α **F** | TCTTGTGGCCCTACTGTGTG | **106bp** |
| **2)Sca-1**α **R** | AGGGTAGGGGCAGGTAATTG |  |
| **3)Sca-1**α **F** | ACACAGCCAGCACAGTGAAG | **118bp** |
| **3)Sca-1**α **R** | ACTCAACAGGGGGACATTCA |  |
| **1)Sox2 F** | AACGCCTTCATGGTATGGTC | **115bp** |
| **1)Sox2 R** | CGGACAAAAGTTTCCACTCC |  |
| **2)Sox2 F** | ACCAGCTCGCAGACCTACAT | **112bp** |
| **2)Sox2 R** | CCTCGGACTTGACCACAGAG |  |
| **3)Sox2 F** | CTTTTGTCCGAGACCGAGAA | **120bp** |
| **3)Sox2 R** | CATGAGCGTCTTGGTTTTCC |  |

Primers are listed in 5′-3′ orientation.
